# F11. TRANSLATIONAL STUDY OF GRIN1, GRIN2A AND 2B GENE EXPRESSION IN PATIENTS WITH SCHIZOPHRENIA AND ANIMAL MODELS

**DOI:** 10.1093/schbul/sby017.542

**Published:** 2018-04-01

**Authors:** Loureiro Camila Marcelino, Corsi-Zuelli Fabiana, Fachim Helene Aparecida, Shuhama Rosana, Joca Sâmia Regiane Lourenço, Menezes Paulo Rossi, Del-Ben Cristina Marta, Louzada-Junior Paulo, Paulo Louzada-Junir

**Affiliations:** 1 University of São Paulo; 2 Ribeirão Preto Medical School, University of São Paulo; 3 School of Pharmaceutical Sciences, University of São Paulo

## Abstract

**Background:**

Changes in glutamatergic system, specifically the ionotropic receptor N-methyl-D-aspartate (NMDAR), are involved in psychosis. NMDARs could be composed of two NR1 and two NR2 subunits. NR1 is one obligatory subunit and is the glycine binding site; and NR2 subunit contain the binding site for the neurotransmitter glutamate and have four different subtypes including NR2A-D. NR1 and NR2A-B are essential subunits of NMDAR, which are encoded by genes Grin1, Grin2A and Grin2B, and have been identified as candidate genes for psychiatric disorders. NMDARs dysfunction disrupts neural excitation and to contribute to the altered brain function underlying, especially in schizophrenia and other psychosis. The aims of this work were 1) to evaluate the expression of Grin1, Grin2A and 2B genes by qPCR of patients with first episode of schizophrenia compared with the siblings and controls; 2) to quantify the NR1 and NR2 subunits plasma concentrations by ELISA; 3) to evaluate the Grin1, Grin2A and 2B gene expression by qPCR in peripheral blood and animals brain tissue.

**Methods:**

Participants will be 30 patients diagnosed with schizophrenia or schizophreniform disorder, including the shorter illness without substance addiction; those participants with siblings who agreed to participate (n = 30) and 30 controls, matched to patients by sex, age and education. Male Wistar rats were kept isolated (n = 10) or grouped (n = 10) from weaning for 10 weeks. After this period the animals were exposed to the Open Field and soon afterwards they were sacrificed, hippocampus and prefrontal cortex (PFC) were dissected to RNA extraction. RT-PCR was performed using probes and TaqMan mastermix to evaluate the mRNA expression. One-way ANOVA with a Bonferroni correction was used for statistical analysis.

**Results:**

Humans: Regarding the glutamatergic system, none of the chosen genes were expressed in the studied sample. Animals: Isolated reared animals presented hyperlocomotion at the two first time bins (0–5 and 5–10 min) in periphery of the arena when compared to the grouped [0–5 min: p = 0.025; 5–10 min: p = 0.002], respectively. Decreased expression of Grin1 (31%), Grin2A (45%) and Grin2B (52%) were found in PFC of isolated animals when compared to grouped (p < 0.05), while no changes were found in the hippocampus.

**Discussion:**

Changes in the expression of essential isoforms (NR1 and NR2) that make up NMDAR in PFC suggest abnormalities of glutamatergic neurotransmission in the pathophysiology of schizophrenia, corroborating recent studies. In addition, this study reinforces the validity of the social isolation rearing model from weaning with a better understanding of the mechanisms of NMDAR hypofunction and the influence of the environment on gene expression in this disorder.

